# Racism, disease, and vaccine refusal: People of color are dying for access to COVID-19 vaccines

**DOI:** 10.1371/journal.pbio.3001167

**Published:** 2021-03-08

**Authors:** Susan M. Reverby

**Affiliations:** 1 Women’s and Gender Studies, Wellesley College, Wellesley, Massachusetts, United States of America; 2 Project on Race & Gender in Science & Medicine, Hutchins Center for African and African American Research, Harvard University, Cambridge, Massachusetts, United States of America

## Abstract

As the vaccines against COVID are slowly becoming available, we need to consider the paradox of why so many people of color are dying from the disease yet cannot get the vaccinations. Focus of concern ought to shift from vaccine refusal to lack of access.

## Teaser

As the vaccines against COVID are slowly becoming available, we need to consider the paradox of why so many people of color are dying from the disease yet cannot get the vaccinations. Concerns focus on vaccine refusal but lack of access is the bigger problem.

In the early 1900s as knowledge of germ theory spread, the *Atlanta Constitution* newspaper in Georgia editorialized that “Germs Know No Color Line,” an early reminder that bacteria do not stay segregated by race [1]. In 2021, unless at least 70% of us get vaccinated or have long-term immunity from having had COVID, we will have proven that “Viruses Know No Color or Ethnicity Lines [2].” Yet widespread beliefs that the virus is no worse than the flu, that masks and social distancing are not necessary, and that the vaccination is more dangerous than the disease will hamper our ability to get to herd immunity. Such beliefs, when they become actions, will prolong the pandemic.

Now that the vaccines are becoming available, albeit too slowly as we try to stop the spread of the virus, we need to consider the paradox of why so many people still seem to be resisting the injections even as they are difficult to get. To explain “vaccine hesitancy” we need a more nuanced read of history and contemporary realities. We must consider why so much focus is on *vaccine refusal*, rather than on the *lack of access* to the vaccine and healthcare in general.

The concern with vaccine hesitancy has been laid primarily at the feet of African American and Latinx communities in the United States. Study after study appears to show that more Black and Brown people, out of proportion to their numbers in the population, are getting sick and dying from COVID-19 compared with whites, yet resisting the vaccinations because of mistrust [3]. Rather than a nuanced analysis for this mistrust of conventional medical care, however, we routinely hear a litany of historical explanations. In Black communities, a holy trinity of medical horror stories are trotted out: Dr. J. Marion Sims’ use of slave women for gynecological experimentation, the 40-year study in Tuskegee of “untreated syphilis in the Male Negro,” or the taking of Henrietta Lacks’ cervical cancer cells to begin the first reproducible cell lines. In the Latinx communities, the explanations focus more on requirements to present government-issued identification, mistrust of government sponsorships given the histories of forced sterilizations and experimentation on Black and Brown bodies, and reliance on herbal remedies [4, 5].

Against this backdrop, many of the news stories on the refusal to wear masks or socially distance tie such actions to political conservatives, libertarians, or some form of toxic masculinity. Yet it is not just white people who get their news from rightwing media outlets, social media, and word of mouth that spread fears of vaccine consequences, or a sense that the government is using the vaccine to harm people, or will implant things to track individuals. Such disinformation and fears have spread through Latinx communities as well [6]. All of these historical and conspiratorial factors matter of course, but in ways not frequently acknowledged. When people of color refer to such historical claims it is often as a way to say that structural racism is real, or I, my family, or community have been subjected to this kind of racist treatment, but when it comes to health care I will explain this experience in historical terms because it sounds less crazy [7]. When mass incarceration, unlawful police actions, and unwarranted immigration raids shape a community’s experience, why should they trust the government? In turn, conspiracy theories have always been fundamental to American politics, infiltrate medical beliefs, and affect health behaviors [8]. The news media’s focus on mistrust or seemingly ridiculous conspiracies, however, ignores the racist structures that shape economic, political, and social realities that lead to health disparities.

The alarming statistics on who is getting the vaccines, and who is not, should shift our attention away from mere mistrust in communities of color and toward the structures of racism that cause that mistrust. Chicago provides a good example. Aware that equity ought to drive the vaccine roll-out, that city’s health department sought to provide local pharmacies with the vaccines. But that well-intentioned effort did not account for the fact that many of the city’s minority communities are “pharmacy deserts” or are populated not by the big chains but by independent stores that do not have the capacity to vaccinate right now. Similarly, when vaccination signups must be done online, lack of access to decent internet connections or even smartphones make it nearly impossible to do [9].

While only a long-term perspective and commitment to public health, primary care, and affordable health insurance can change some of the underlying structural problems that shape the pandemic, there is much we can do now: acknowledge that the mistrust is realistic, keep an equity lens in full view as the vaccines are rolled out, consider deploying vaccinators to local churches and independent pharmacies, keep explaining why the vaccine is safe and effective and be transparent if there are problems, make sure that immigration police are kept out of vaccination centers, press for vaccination in the prisons, or better yet decarceration. We may be in a biological battle with the virus, but winning will require more than medical research prowess alone. We must harness social and political tools to dismantle the structural barriers that perpetuate deadly health disparities.
